# Evaluation of Self-Nanoemulsifying Drug Delivery Systems (SNEDDS) for Poorly Water-Soluble Talinolol: Preparation, *in vitro* and *in vivo* Assessment

**DOI:** 10.3389/fphar.2019.00459

**Published:** 2019-05-02

**Authors:** Mohsin Kazi, Mohammed Al-Swairi, Ajaz Ahmad, Mohammad Raish, Fars K. Alanazi, Mohamed M. Badran, Azmat Ali Khan, Amer M. Alanazi, Muhammad Delwar Hussain

**Affiliations:** ^1^Department of Pharmaceutics, College of Pharmacy, King Saud University, Riyadh, Saudi Arabia; ^2^Department of Clinical Pharmacy, College of Pharmacy, King Saud University, Riyadh, Saudi Arabia; ^3^Pharmaceutical Biotechnology Laboratory, Department of Pharmaceutical Chemistry, College of Pharmacy, King Saud University, Riyadh, Saudi Arabia; ^4^Department of Pharmaceutical and Biomedical Sciences, College of Pharmacy, California Health Sciences University, Clovis, CA, United States

**Keywords:** self-nanoemulsifying drug delivery systems (SNEDDS), lipid based formulations (LBFs), lipid formulation classification system (LFCS), talinolol, *in vitro* dissolution, RBC toxicity, gut permeability, oral bioavailability

## Abstract

**Objective:**

The aim of this study was to investigate the *in vitro* and *in vivo* performance of self-nanoemulsifying drug delivery systems (SNEDDSs) of talinolol (TAL), a poorly water-soluble drug.

**Methods:**

Self-nanoemulsifying drug delivery systems of TAL were prepared using various oils, non-ionic surfactants and/or water-soluble co-solvents and assessed visually/by droplet size measurement. Equilibrium solubility of TAL in the anhydrous and diluted SNEDDS was conducted to achieve the maximum drug loading. The *in vitro* dissolution experiments and human red blood cells (RBCs) toxicity test, *ex vivo* gut permeation studies, and bioavailability of SNEDDS in rats were studied to compare the representative formulations with marketed product Cordanum^®^ 50 mg and raw drug.

**Results:**

The results from the characterization and solubility studies showed that SNEDDS formulations were stable with lower droplet sizes and higher TAL solubility. From the dissolution studies, it was found that the developed SNEDDS provided significantly higher rate of TAL release (>97% in 2.0 h) compared to raw TAL and marketed product Cordanum^®^. The RBC lysis test suggested negligible toxicity of the formulation to the cells. The *ex vivo* permeability assessment and *in vivo* pharmacokinetics study of a selected SNEDDS formulation (F6) showed about four-fold increase in permeability and 1.58-fold enhanced oral bioavailability of TAL in comparison to pure drug, respectively.

**Conclusion:**

Talinolol loaded SNEDDS formulations could be a potential oral pharmaceutical product with high drug-loading capacity, improved drug dissolution, increased gut permeation, reduced/no human RBC toxicity, and enhanced oral bioavailability.

## Graphical Abstract

Self-nanoemulsifying drug delivery systems (SNEDDS) of talinolol (TAL) were developed using lipid formulation classification systems (LFCS) that improved the solubility and released the drug completely in dissolution media. *In vitro* toxicity studies with human red blood cells (RBC) showed that the SNEDDS formulations were non-toxic to human RBC. The oral bioavailability of TAL was significantly enhanced by the SNEDDS formulation in rats. Schematic Diagram: *In vivo* processes occurring after ingestion of SNEDDS formulations developed based on LFCS.

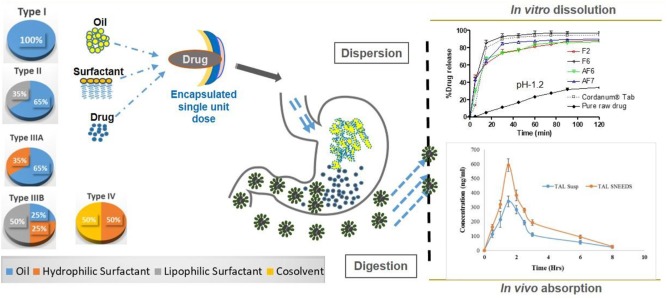

## Introduction

**FIGURE 1:**
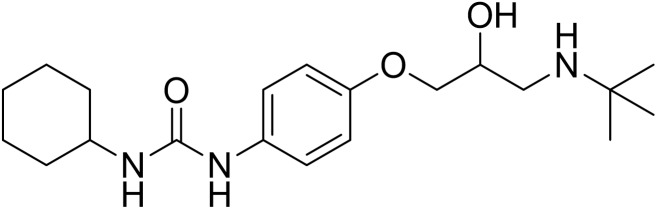
Chemical structure of talinolol (TAL, M.W: 363.5, pKa: 9.4).

Talinolol (TAL) [1-(4-cyclohexylureido-phenoxy)-2-hydroxy- 3-tert-butylaminopropane] (Figure 1) is a long-acting, highly selective β_1_ -adrenergic receptor antagonist which is incompletely absorbed in human from the upper small intestine (Schwarz et al., 2000; Siegmund et al., 2003). TAL (MW 363.49) is a moderately lipophilic molecule (log P: 3.2) with ionizable groups (pKa: 9.4). The solubility of TAL is pH dependent with low solubility at higher pH (0.02 mg/ml at pH 7.4, 37°C) (Trausch et al., 1995a). It has moderate lipophilic properties compared with other β-blockers (Trausch et al., 1995a; Pathak et al., 2010). The commercial product of TAL (Cordanum^®^) has been frequently used in Germany and Eastern Europe for almost five decades for the treatment of anxiety, hypertension, angina pectoris, cardiac arrhythmias, glaucoma, and migraine headaches with oral doses of 50 to 300 mg per day (Wetterich et al., 1996; Chiou et al., 2001; Awadallah et al., 2003).

Talinolol has a variable oral bioavailability of 36 to 69% in humans with physicochemical properties suitable for lipid-based self-nanoemulsifying formulations (Terhaag et al., 1992; Trausch et al., 1995a; Elgart et al., 2013). The reduced and variable bioavailability may be attributed to precipitation of TAL in the gastro intestinal tract (GIT), incomplete and erratic absorption, and P-gp mediated efflux transport in the intestine (Gramatte et al., 1996). It is essential to reduce the precipitation and maintain TAL solubilized in GIT to ensure its absorption in to the systemic circulation (Mohsin et al., 2009; Ghai and Sinha, 2012). Solubility and dissolution is primarily the rate limiting factors for oral bioavailability of many drugs. There have been a variety of formulation strategies designed over the past two decades to improve drug solubility to enhance the rate and extent of drug absorption from the GIT (Shahba et al., 2012, 2016, 2017; Devraj et al., 2013; Mohsin et al., 2016).

Self-emulsifying drug delivery systems which belongs to lipid based formulations (LBFs), have shown to improve the slow and incomplete drug dissolution, and facilitate the formation of the highly solubilized phase of drug for enhanced drug absorption. Also, the self-emulsifying formulations can be easily filled in soft and hard gelatin capsules due to their anhydrous nature (Pouton, 1985; Strickley, 2004).

Self-emulsifying formulations are isotropic mixtures of an active drug compound in a combination of lipids, surfactants, and water soluble co-solvents that produce ultrafine emulsions upon gentle agitation in aqueous phase, such as the upper GI lumen content (Fatouros et al., 2007; Kale and Patravale, 2008). Generally, self-emulsifying formulations are categorized as self-emulsifying (SEDDS), self-microemulsifying (SMEDDS) and self-nanoemulsifying drug delivery systems (SNEDDSs). SEDDS, SMEDDS, and SNEDDS can be differentiated basically according to their size of globules upon aqueous dispersion (Pouton, 2000; Pouton and Porter, 2008). A lipid formulation classification system (LFCS) based on the composition was developed which categorized the LBF into four different types (Pouton, 2000). The LFCS explains the formation of different types of self-emulsifying formulations in a very simple way based on their types and compositions. Briefly, Type I formulations represent 100% pure oil (surfactant free) as component. Types II and IIIA systems contain water insoluble surfactants (HLB < 10) with different % oil in the formulation (Type II contain 60–80% oil and Type IIIA contain 40–60% oil). Type IIIB formulations contain water soluble surfactant and oil (20–50% oil), whereas Type IV formulations contain only water soluble surfactant/cosolvent without oil. In board terms, the Type II and/or Type IIIA formulations spontaneously forms SEDDS emulsions (translucent) with a droplet size ranging from 250 nm to 1.0 μm. SMEDDS refers to the formulations which form transparent microemulsions (oil-in-water/water-in-oil) with a particle diameter of 50 nm or less, belong to Type IIIB and or Type IV. On the other hand, SNEDDS is a relatively newer term representing the droplet size between 20 and 200 nm, provides also transparent appearance. The drug encapsulated as liquid dosage forms can be in a thermodynamically and kinetically stable form as SNEDDS (Kang et al., 2004; Elgart et al., 2013).

Self-nanoemulsifying drug delivery systems is an efficient, elegant, and more patient compliant formulation approach for poorly water soluble drugs. It may enhance drug solubility, dissolution behavior in the GIT, gut permeability and thus may increase the absorption of the poorly water soluble model drug, TAL (Elgart et al., 2013; Shakeel et al., 2016). The purpose of the current study was to enhance bioavailability of TAL by designing suitable SNEDDS formulations.

## Materials and Methods

### Materials

Talinolol (purity > 99.2%) was purchased from Alfa Aesar (Ward Hill, MA, United States). Miglyol 812 (medium chain triglyceride, M812), Imwitor 988 (medium chain mono- and di-glycerides, I988), Imwitor 308 (medium chain monoglycerides, I308) were kindly supplied by Sasol Germany GmbH (Werk, Witten, Germany). HCO-30 (hydrogenated castor oil, HLB = 11) and POE-6-sorbitan monooleate (TO-106V, HLB = 10.5) were obtained from Nikko Chemicals (Tokyo, Japan). Capmul MCM (CMCM) was obtained from Abitec Corporation, Janesville, WI, United States, Coconut oil fatty acid (COFA). Softigen 767 (S767) was purchased from Cremer oleo GmbH & Co. (Hamburg, Germany). Phosal 25 MCT (P25MCT) was obtained from Lipoid GmbH (Steinhausen, Switzerland). Transcutol (TC) was kindly supplied by Gattefossé (Lyon, France). Talinolol commercial tablets (Cordanum^®^ 50 mg) were a gift from AWD, pharma GmbH & Co., KG (Dresden, Germany). Capsule of plant origin with a size of (00) were obtained from Qualicaps, Co., Ltd. (Japan). Simulated intestinal fluid (SIF) powder (bile salt/phospholipid) was purchased from Biorelevant.com, Ltd., London, United Kingdom. Porcine pancreatin (8_USP specifications activity) was purchased from Sigma Chemical, Co., St. Louis, MO, United States. Milli-Q grade water was obtained from a Milli-Q Integral Water Purification System (Millipore, Bedford, MA, United States).

### Methods

#### Design of Self-Nanoemulsifying Lipid Formulations (SNEDDSs)

The self-emulsifying formulations were prepared using different natural/semi synthetic oils, lipophilic/hydrophilic surfactants and water-soluble cosolvents. Different types of excipients were made to establish a wide range of self-emulsifying compositions. A whole range of formulations and alternative formulations (F and AF) from LFCS Types I to IV were prepared and studied using 10 components; M812, COFA, I988, I308, P25MCT, CMCM, S767, TO-106V, HCO-30, TC, and by changing one excipient at a time.

#### Visual Assessment Method of the Formulations

A visual assessment method was used to determine the self-emulsification properties of the formulation (Craig et al., 1995; Kommuru et al., 2001). For sample preparation, a 100 μl of each formulation was diluted with 10 ml of water in a 20 ml screw capped glass vial (1:100 dilutions) and agitated gently for 1 min at room temperature. For self-emulsifying efficiency, visual assessment method initially is able to minimize the excess usage of chemicals. The visual assessment was observed/recorded immediately after preparation and for several months for each sample.

#### Determination of Droplet Size, Polydispersity Index (PDI), and Zeta Potential Values

The droplet size, polydispersity index (PDI) and the zeta potential (surface charge) of all the representative diluted “F” and “AF” systems were measured by a laser light diffraction analysis technique using Zetasizer Nano (Model ZEN3600, Malvern, United Kingdom) particle sizing systems. The formulations were diluted at a ratio of 1:1000 *v/v* with water and mixed well for 1 min. The diluted samples were transferred into cuvettes and 10 repeated readings were performed for each sample. Samples from each three (3) separate batches of a formulation were used to determine the droplet size and zeta potential values.

#### Transmission Electron Microscopy Analysis

For the analysis of transmission electron microscopy (TEM), samples were properly diluted with water. A drop of diluted sample was placed on 300 mesh carbon coated copper grid (Ted pella). Grid was left for 5 min to settle down the droplets. Excess of liquid was removed using filter paper and grid was left to air dry. Then a drop of 1% phosphotungustic acid in water was added to the grid. Phosphotungustic acid works as negative stain. Again this left for 5 min to settle down and dried as previous. Finally, dried grid was visualized under Jeol TEM, JEM1010 (Japan) at an operating voltage of 80 kV. Images of the vesicles were captured using iTEM software and olympus Megaview G2 top mounted camera.

#### Equilibrium Solubility Studies of TAL

##### Anhydrous formulations

Anhydrous formulation represents the pre-concentrate, which can have maximum drug loading capacity. The solubility of TAL in the formulations was determined using the common shake flask method. Samples were prepared by adding an excess amount of TAL to the formulations followed by subsequent shaking with the vortex mixer for 3 min at room temperature (25 ± 2°C) to ensure adequate mixing. After incubation in a dry heat incubator at 37°C for 7-days period; the samples were removed for solubility determinations. The samples were placed in 1.5 mL eppendorf tubes and centrifuged (at 13000 rpm for 10 min) to separate excess solid drug from the dissolved drug. Then, an aliquot of the supernatant was taken by weight (approximately 50 mg) and diluted in 25 ml of acetonitrile. The amount of TAL solubilized was analyzed using a validated (UHPLC) method developed in our laboratory. Three replicate samples were considered for analysis of each formulation system.

##### Diluted formulations (99% or 100 times’ dilution)

An effective way to estimate the likely fate of the drug upon dispersion or dilution with water is to examine its solubility in the maximum diluted formulation (Mohsin and Alanazi, 2012). By this method one can predict how much TAL can be loaded in the anhydrous formulation and intended dose to be diluted in the average volume of gastric fluids. The 99% diluted formulations were prepared using water as diluent, each 1 mL of the anhydrous formulation (without any drug) was mixed with 99 mL of water (100 times dilution) and vortexed to ensure the formulation was well-mixed. In addition, TAL solubility was performed in water, and bile salt/phospholipid micellar solutions. Samples were prepared by adding an excess amount of TAL to the diluted formulations and shaken with the vortex mixer occasionally while keeping in a dry heat incubator at 37°C for a 7-day period, this was needed to see how much TAL remained in the solution after 100 times dilution. After 7 days, the solubility of TAL was determined in the aqueous and 99% diluted formulations by similar method used for the anhydrous formulations above.

#### *In vitro* Dissolution Studies

The most efficient SNEDDS formulations were selected according to the preliminary assessment studies and optimal solubilization capacity of the TAL. The dissolution studies of the selected SNEDDS formulations were performed using an automated dissolution tester (LOGAN Instrument, Corp., Somerset, NJ, United States) connected to an automated sample collector (SP-100 peristaltic pump, Somerset, NJ, United States). The standard USP II (Model UDT-804, LOGAN, Inst. Corp., United States) paddle method was used at 37 ± 0.5°C. The volume of the dissolution medium was 500 ml MilliQ water with pH 1.2 and 6.8 to simulate stomach and intestinal media respectively (Kazi et al., 2017). The rotation speed of the paddle was adjusted to 50 rpm. The drug loaded formulations (encapsulated in hard gelatin capsules, size “00”) were dropped into dissolution medium using capsule sinkers to prevent the capsules from floating in the medium. The capsule was filled with SNEDDS (equivalent to 50 mg of TAL) and the commercial tablet Cordanum^®^ contained 50 mg of TAL.

An aliquot (3 mL) of the sample was collected periodically after 0, 5, 15, 30, 45, 60, 75, 90, and 120 min and replaced by a freshly prepared equivalent volume of dissolution medium. The withdrawn samples were filtered through a 0.20 μm syringe filter (CHROMAFIL^®^ PTFE-20/25) prior to TAL analysis with a UHPLC system (Dionex, Ultimate 3000, Thermo Scientific, United States) using the chromatographic conditions reported earlier (32). The dissolution studies were carried out in triplicate.

#### *In vitro* Lipolysis Tests (Analysis of Post-digested Products)

For each lipolysis test, 250 mg of TAL loaded formulation was dispersed to 9 mL of a digestion buffer under fed (101 mM NaOH, 144 mM Glacial acetic acid, 203 mM NaCl, pH 5.0) and fasted (10.5 mM NaOH, 28.6 mM NaH_2_PO_4_.H_2_O, 105.9 mM NaCl, pH 6.5) conditions, respectively. SIF powder containing taurocholate (bile salt) and lecithin (phospholipid) in a 4:1 molar ratio (Galia et al., 1998) were included in the digestion mixture, which is the ratio secreted in bile. The lipid formulations were emulsified in the mixed micellar solutions prior to enzyme addition by stirring continuously for 10 min in the thermostatic jacketed glass reaction vessel. Experiments were performed at 37°C and 1 ml of pancreatin extract containing 800 tributyrin units of pancreatic lipase was then added to initiate lipolysis (Mohsin et al., 2009). Lipolysis was allowed to continue for 30 min using a pH-stat titration unit (902 Titrando by Metrohm AG, Switzerland), which maintained the constant pH at 6.8. The fatty acids produced on lipolysis were titrated with 0.2 M NaOH for all the formulations.

At the end of each digestion experiment, three samples of 2 mL of digestion mixtures were transferred into polyallomer centrifuge tubes and 20 μL of 4-bromophenylboronic acid was added to each sample to prevent further lipolysis. Samples were then ultra-centrifuged (model Optima^TM^ MAX-E-100K; Beckman, Palo Alta, CA, United States) at 80000 rpm for 50 min at 4°C utilizing a SW-60 swinging bucket rotor to separate the different digestion phases. After ultracentrifugation, the formulation digests were separated into an aqueous phase and a precipitated pellet phase. In this study, only two of the LFCS Types IIIB and IV representative formulations were used, where Type IV was explored as an example of the extremely hydrophilic formulation to compare their performance under the same experimental conditions. Sample obtained from each of the separated phases was analyzed for drug content by UHPLC as described previously.

#### *Ex vivo* Permeation Studies

The male Wistar rats (weighing 200–250 g) were housed in a room temperature and humidity (55% air humidity) with free access to standard diet and water. The rats were fasted overnight, but provided with water only before the study. Animals were sacrificed by spinal dislocation. The small intestine was directly detached after a sacrifice by cutting across the duodenal upper end and the lower end of the ileum and stripping the mesentery. The small intestine was washed out carefully with Kreb’s Ringer phosphate buffer (KRPB) solution using a syringe with a blunt end. The clean intestinal sacs were cut into 6.0 ± 0.5 cm. The selected SNEDDS (F6) were dispersed with 1 mL of KPRB. A suspension of marketed tablet and TAL powder (control) was also dispersed in 1 ml of KRPB at same drug concentration. Six sacs were filled via a blunt needle with SNEDDS (F6) and TC solution (equivalent to 4 mg of the drug) and the other six sacs were filled with an equivalent amount of the tablet and powder suspensions. The two sides of the intestine were tied tightly with a thread. Each sac was placed in a glass test tube containing 10 mL of KRPB solution. The entire system was maintained at 37°C in a shaking water bath operated at 100 rpm and aerated using a laboratory aerator. Samples were withdrawn from outside the sac and the medium was totally replaced by fresh medium over 2 h. Samples were analyzed by UHPLC.

The permeability of TAL was obtained by plotting the cumulative amount of drug permeated through the sac versus time (Iglicar et al., 2009). An apparent permeability coefficient (Papp) of TAL was calculated according to the equation:

$$Papp(cm/\sec)=(dQ/dt)/\left(A/C_{0}\right)$$

Where, the dQ/dt is the drug permeation rate from the intestinal membrane, A is the cross-sectional area of the tissue, and C_o_ is the initial TAL concentration in the donor compartment at t_0_ (Iglicar et al., 2009).

#### RBC Lysis Test

*In vitro* acute toxicity of TAL loaded SNEDDS (F6) was studied by employing red blood cell (RBC) lysis test (Khan et al., 2012). Briefly, blood from healthy human was taken in anticoagulant solution and centrifuged at 1000 *g* for 15 min at 4°C. Supernatant containing the buffy coat and plasma was discarded. The pellet containing RBC was washed and diluted with isotonic buffer (10 mM phosphate buffer, 150 mM NaCl) and 50% hematocrit was prepared. The aliquots of RBC suspension were incubated at 37°C for 1 h with 200 μg/ml of each tested formulation. After 1 h, the aliquots were centrifuged at 1500 *g* and supernatant was evaluated for released hemoglobin by UV – Visible spectroscopy (λmax = 576 nm).

#### Bioavailability Studies

Healthy male Wistar rats (200 ± 20 g) were obtained from the Central Animal House Facility of the College of Pharmacy, King Saud University (Riyadh, Saudi Arabia) and maintained in plastic animal cages; six animals were housed per cage with a 12-h light/dark cycle, at 25 ± 2°C. This study was carried out in accordance with the principles of National Institute of Health Guide for the Care and Use of Laboratory Animals (NIH Publications No. 80–23; 1996). The study was approved by the animal facilities guidelines from the Ethical committee of Experimental Animal Care Center, College of Pharmacy, King Saud University (Clearance No. 5698; December, 2016). The animals were acclimatized to laboratory conditions for 1 week prior to experiments. The animals were divided into two groups (total 12 rats; 6 rats in each group) and fasted for 12 h before the experiment to avoid any food–drug interaction, but were provided *ad libitum* access to water. The animals were provided normal diet after 1 h of dosing.

The bioavailability of a selected SNEDDS formulation of TAL [F6, I308: TC (1:1)/HCO-30 (1/1)] was compared with pure TAL powder. Both, SNEDDS formulation of TAL (diluted with normal saline, 1:10 ratio) and pure TAL powder (suspended in normal saline, 17.5 mg/ml) were administered orally at a dose equivalent to 10 mg/kg of TAL. Blood samples (200 μL) were collected from fossa orbital’s vein under light anesthesia to minimize pain to the animal (Raish et al., 2019). The samples were collected at 0.0, 0.5, 1, 1.5, 3, 4.5, 6, and 8 h after drug administration in heparinized tubes. Blood volume was replaced with saline after withdrawal of each sample.

Plasma was separated from the blood samples by centrifugation at 2500 × *g* for 10 min and stored at -20°C until analysis.

##### TAL plasma samples analysis

Liquid–liquid extraction (LLE) procedure was used for the extraction of TAL from the rat plasma. The plasma samples (≈100 μL) were transferred into 1.5 mL Eppendorf tubes. The internal standard solution (25 μg/ml in methanol) was added to the plasma sample and vortexed. Plasma protein precipitation was carried out by addition of 1.0 ml methanol to the plasma sample. The sample tubes were vortexed for 5 min (Trausch et al., 1995b) and centrifuged for 10 min at 2500 × *g*. The supernatant (organic layer) of the sample was transferred into clean centrifuge tubes and was evaporated to complete dryness under nitrogen gas at 45–50°C. The dry residue was then reconstituted with 125 μl of mobile phase, and vortexed. The concentration of TAL in the reconstituted residue was determined by a modified UHPLC method reported earlier (Weng et al., 2014).

##### Pharmacokinetic data analysis

A non-compartmental pharmacokinetic analysis method was used to investigate the pharmacokinetic behavior of TAL. Microsoft Excel was used to calculate the pharmacokinetic parameters from the experiments. Area under plasma concentration-time curve (AUC) was calculated with the help of the linear trapezoid method. The relative bioavailability of the representative SNEDDS to the control was calculated as follows: Relative bioavailability % = AUC_SNEDDS/_AUC_control_. The apparent elimination half-life (T_1/2_) was determined as 0.693/K_el_. The maximum plasma concentration (C_max_) and time to maximum concentration (T_max_) after oral administration were determined directly from the concentration versus time curve.

#### Statistical Analysis

##### *In vitro* and *in vivo* pharmacokinetic data

Differences in pharmacokinetic parameters (e.g., CL/F, V_ss_, AUC_0-∞_) of SNEDDS formulation of TAL and control TAL powder was assessed by paired *t*-test using GraphPad Prism^®^ (4.5 version) for Windows (San Diego, CA, United States). The one-way ANOVA test followed by *post hoc* analysis was employed to compare the *in vitro* dissolution profiles and *ex vivo* permeability studies. *p*-Values ≤0.05, ≤0.001, ≤0.0001 were considered as statistically significant for all the data obtained from dissolution, *ex vivo* permeability and pharmacokinetic studies.

## Results and Discussion

### Design of Self-Nanoemulsifying Lipid Formulations (SNEDDSs)

The Self-emulsifying nature of the various formulations upon aqueous dispersion showed different degrees of transparency ranging from clear to whitish milky suspension of the formulations. The formulations were optimized on the basis of their self-emulsification efficiencies. The compositions of the formulations and the appearance upon aqueous dispersions are presented in Table 1. The most interesting formulation systems, which were able to produce nanoemulsions (SNEDDS), belong to the LFCSs Types IIIB and IV. These SNEDDS (Table 1) components were developed using polar glycerides and water soluble surfactant and cosolvent.

**Table 1:** The assessment of various formulations within lipid formulation classification systems (LFCSs) under specific self-emulsification condition visually.

LFCS	No.	Composition	Appearance	Suitable as SNEDDS
Type I	F1	COFA	Turbid and non-dispersed	Fail
	AF1	M812	Turbid and Poorly dispersed	Fail
Type II	F2	COFA:I988(7:3)/TO106V(1/1)	Milky/Turbid	Fail
	AF2	M812:I988(7:3)/TO106V(1/1)	Milky/Turbid	Fail
	F3	P25MCT/TO106V(1/1)	Milky/Turbid	Fail
	AF3	S767/ TO106V(1/1)	Bluish	Pass
Type IIIA	F4	CoFA:I988(7:3)/HCO-30(1/1)	Bluish	Pass
	AF4	M812:I988(7:3)/HCO-30(1/1)	Bluish	Pass
Type IIIB	F5	I308/HCO-30(1/1)	Bluish	Pass
	AF5	CMCM/HCO-30(1/1)	Bluish	Pass
	F6	I308:TC(1:1)/HCO-30(1/1)	Transparent	Pass
	AF6	CMCM:TC(1:1)/HCO-30(1/1)	Transparent	Pass
Type IV	F7	HCO-30	Transparent	Pass
	AF7	TC	Transparent	Pass


*The systems show the composition of excipients used in each formulation and their appearances upon aqueous dispersion (“F” and “AF” denoted “Formulation” and Alternative Formulation” respectively).*

### Visual Assessment Method of the Formulations

**FIGURE 2:**
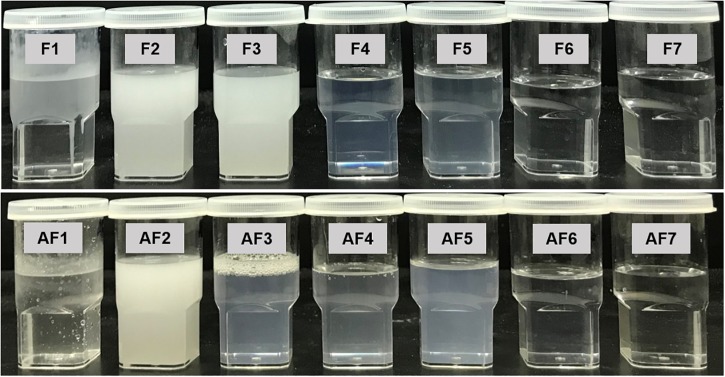
Typical physical appearance of the “Formulations” (F) and the “Alternative Formulations” (AF) after immediate aqueous dispersion with water. The samples used in the experiment were aqueous dispersions of the “drug loaded” formulations.

A visual assessment method was used as a guideline for evaluating the miscibility, homogeneity and the appearance of the formulations. Visual assessment of all Types III–IV (F4–F7, AF4–AF7) formulations confirmed that the emulsion formed upon dilution with water was physically stable for several months. Both lipophillic and hydrophilic surfactants with HLB 10–14 were able to promote self-emulsification of pure oil. However, the resulting emulsions (F2, F3, AF2, Type II) appeared crude (visually turbid/milky, higher droplet size, with lipophilic surfactant TO106V) (HLB number 10.5) (Figure 2), whereas it was a much better appearance with hydrophilic surfactants HCO-30 (HLB number 11) for Types IIIA, IIIB, and IV. They provided fine (bluish/transparent systems without any visible particulates), uniform emulsion droplets which are more likely to empty rapidly from the stomach due to their fine particles upon dispersion. The alternative formulation (AF3) produced better appearance and lower droplet sizes compared to F3 within Type II systems. Pure fatty acid and glyceride oils (e.g., COFA- F1 and M812- AF1) showed very poor self-emulsification properties and did not form SNEDDS. They formed oil globules non-dispersed in aqueous media along with turbid appearances.

The formulations (Type II) containing lipophilic components (M812, P25 MCT, I988, TO106V), i.e., F2, AF2, F3 were milky and or turbid in appearance upon aqueous dispersion and also were not suitable as a SNEDDS formulation (Failed as SNEEDS). However, when the glycerides oils were mixed with water soluble surfactant (surfactant of hydrogenated castor oil) and cosolvent, the representative formulations were considered as SNEDDS (Passed as SNEDDS, Table 1) and their appearances were either transparent or bluish with homogeneous dispersion (e.g., F4-F7 and AF3-AF7).

The overall study on visual assessment of formulations suggest that excipient selection during formulation development study influences performance of the pharmaceutical drug product such as stability, bioavailability, and manufacturability (Craig et al., 1995).

### The Effect of Formulation Hydrophilicity on the Droplet Size

**FIGURE 3:**
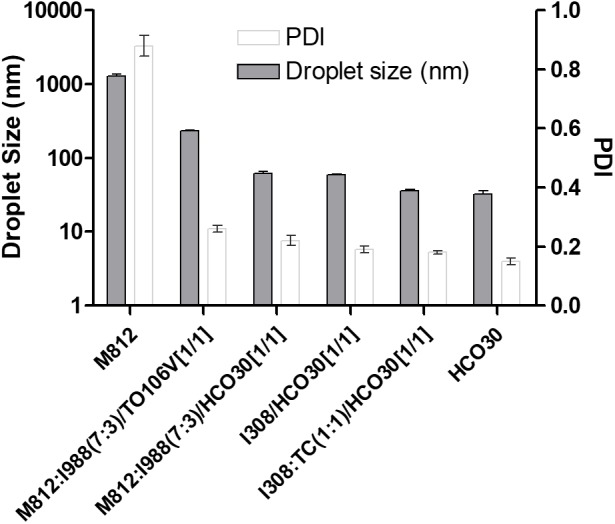
The effect of hydrophilic compositions on the particle size and PDI of lipid based formulations (LBFs) for TAL. Formulations represent M812-Type I (AF1), M812:I988 (7:3)/TO106V (1/1)-Type II (AF2), M812:I988 (7:3)/HCO30(1/1)-Type IIIA (AF4), I308/HCO30(1/1)-Type IIIB (F5), I308:TC (1:1)/HCO30(1/1)-Type IIIB (F6), and HCO 30, Type IV (F7). Data are mean ± SD (*n* = 3), Left Y-axis is presented in Log 10 scale.

The effect of water soluble components in various “F” and “AF” formulations on the variation of droplet sizes and PDI are clearly shown in Figure 3. The Type IV formulation, which contained the highest concentration of water soluble components produced the lowest droplets (F7, 32.57 nm) upon dilution with water. The largest droplet sizes were formed with Type I formulation (AF1, 1274 nm, PDI- 0.88) as it contained 100% oil and the formulation was poorly dispersed in water. As Type II (AF2) formulation contained 0–20% water soluble materials, the droplet sizes considerably dropped down to 231.42 nm (*p* < 0.005) with lower PDI value of 0.22 (monodispersed). However, due to the increased amount of water soluble materials (40–80%) in Types IIIA and IIIB formulations, the droplet sizes were decreased considerably to the ranges of 62–35 nm (*p* < 0.005). Among all formulation systems, comparably F6, was found to be the most stable system with small droplet size (35.99 nm) and lower PDI value (0.18) for TAL upon aqueous dilution.

In addition, the zeta-potential values of all the formulations of TAL were found in between -1.0 and -44.0 mV. However, the zeta potential (surface charge) values of the formulations from -15.0 to -32.0 were expected to be highly stable with no aggregation of droplets in the continuous phase (Table 2). This might be due to higher concentration of the non-ionic surfactant that may cause better self-emulsifying systems fabricating a negatively charged interface around the oil droplets, thereby enhancing the stability of formulations.

**Table 2:** The particle size, PDI, and zeta potential (surface charge) of TAL loaded various LFCS formulations.

No.	Composition	Size nm	PDI	Zeta
F1	COFA	3423 ± 344	1.00	-17.73 ± 5.86
AF1	M812	1274 ± 34	0.88	-30.00 ± 8.36
F2	COFA:I988(7:3)/TO106V(1/1)	253.73 ± 7.99	0.42	-34.20 ± 1.6
AF2	M812:I988(7:3)/TO106V(1/1)	231.42 ± 3.61	0.22	-31.33 ± 0.9
F3	P25MCT/TO106V(1/1)	150.20 ± 3.96	0.43	-44.27 ± 3.53
AF3	S767/TO106V(1/1)	45.11 ± 0.16	0.40	-30.10 ± 0.70
F4	CoFA:I988(7:3)/HCO-30(1/1)	57.53 ± 11.54	0.45	-1.21 ± 0.36
AF4	M812:I988(7:3)/HCO-30(1/1)	36.77 ± 0.77	0.42	-4.33 ± 1.01
F5	I308/HCO-30(1/1)	62.13 ± 5.55	0.39	-8.49 ± 0.39
AF5	CMCM/HCO-30(1/1)	47.37 ± 0.85	0.43	-16.13 ± 0.91
F6	I308:TC(1:1)/HCO-30(1/1)	35.99 ± 4.64	0.19	-22.90 ± 0.44
AF6	CMCM:TC(1:1)/HCO-30(1/1)	10.04 ± 0.02	0.26	-19.55 ± 6.42
F7	HCO-30	32.57 ± 4.63	0.30	-13.26 ± 5.09
AF7	TC	516.6 ± 315.63	1.00	-17.37 ± 2.87


*Formulations were dispersed in water at 1:1000 dilutions level (% w/v). Data represented as mean ± SD, *n* = 3–6.*

This result is consistent with the previous reports (Mohsin et al., 2016). The decrease in the droplet size might be the result of more surfactant being available to stabilize the oil-water interface. It is more likely with SNEDDS formulations that the larger the surface area, the faster and complete are the absorption rate of drugs (Mohsin et al., 2016). Furthermore, the decrease in the droplet size represents the formation of a unique close-packed surfactant film at the oil-water interface (Table 2), there by stabilizing the oil droplets (Gershanik and Benita, 2000; Pouton, 2000).

### Transmission Electron Microscopy

**FIGURE 4:**
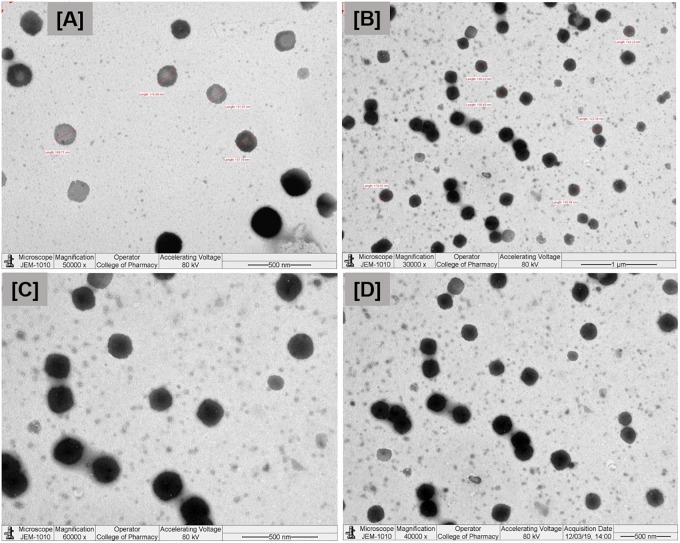
Transmission electron microscopy (TEM) images of the droplets at different nanoscale ranges **(A–D)** of the optimized self-nanoemulsifying formulation. Formulation represents I308:TC (1:1)/HCO30(1/1)-Type IIIB (F6).

Transmission electron microscopy images of diluted representative SNEDDS formulation (F6) is shown in Figure 4. The droplet size was found to be around 150 nm, having size distribution similar to the results obtained by the Zetasizer Nano particle sizing systems. The droplet size upon dispersion determined by TEM imaging techniques also give information on particle shape, which can affect dissolution of the drug. This factor is often ignored in many emulsion development assays, which assume particle shapes as spherical.

### Equilibrium Solubility Studies

#### Anhydrous Formulations

The main objective of this experiment was to increase the solubility of TAL in SNEDDS formulations, thus to reduce the dose and improve the oral bioavailability. The equilibrium solubility of TAL in various LFCS formulations is presented in Table 3. Solubility studies clearly indicated that TAL has high solubility in the formulations containing higher fatty acids. The solubility was also higher in Types IIIB and IV formulations (hydrophilicity increased). Amongst the various formulations, F1, F2, F6, AF6, and AF7 were found to solubilize higher amounts of TAL. The drug loading was found to be comparatively higher in the formulations containing fatty acid (F1) and water soluble cosolvent (AF7).

**Table 3:** The equilibrium solubility of TAL in various LFCS “Formulations” and “Alternative Formulations” at anhydrous and 99% dilutions.

No.	Composition	Solubility (mg/g)	
		
		Anhydrous formulation	99% diluted formulation
F1	Coconut oil FA (COFA)	346.81 ± 0.68	1.36 ± 0.01
AF1	M812	0.60 ± 0.03	0.29 ± 0.02
^∗^F2	CoFA:I988(7:3)/TO106V(1/1)	44.05 ± 0.15	0.76 ± 0.13
AF2	M812:I988(7:3)/TO106V(1/1)	25.31 ± 0.02	0.88 ± 0.02
F3	P25MCT/TO106V(1/1)	14.94 ± 0.91	0.68 ± 0.13
AF3	S767/TO106V(1/1)	19.57 ± 0.01	0.85 ± 0.01
F4	CoFA:I988(7:3)/HCO-30(1/1)	32.17 ± 0.06	1.27 ± 0.27
AF4	M812:I988(7:3)/HCO-30(1/1)	10.23 ± 0.27	1.02 ± 0.01
F5	I308/HCO30(1/1)	35.03 ± 0.05	1.72 ± 0.01
AF5	CMCM/HCO30(1/1)	29.02 ± 0.18	1.64 ± 0.05
^∗^F6	I308:TC(1:1)/HCO30(1/1)	51.41 ± 2.01	1.76 ± 0.01
^∗^AF6	CMCM:TC(1:1)/HCO30(1/1)	48.79 ± 0.30	1.78 ± 0.04
F7	HCO30	9.06 ± 0.09	1.03 ± 0.01
^∗^AF7	TC	147.35 ± 0.60	2.16 ± 0.02
Media	Water	0.23 ± 0.01	N/A
Media	FaSSIF	2.16 ± 0.02	N/A
Media	FeSSIF	4.53 ± 0.01	N/A


*Data are mean ± SD, *n* = 3–6 (^∗^selected for dissolution studies).*

#### Equilibrium Solubility in Aqueous and 99% Diluted Formulation

An essential consideration while designing self-emulsifying formulation is avoiding precipitation of the drug upon dilution in the gut lumen *in vivo*. Therefore, selection of the suitable oil/surfactant components are crucial for the model drug, which can maintain the drug in solubilized form upon aqueous dilution. The solubility of TAL in aqueous solutions and 99% diluted F and AF formulations depicted in Table 3 confirm that F1 (Coconut oil FA) has an ability of dissolving very large amounts of the TAL in anhydrous pre-concentrate but the formulation appeared immiscible upon aqueous dispersion (large oil globules with low drug solubility), thus it was avoided.

The key mechanism by which lipid-based formulations improve the bioavailability of poorly water soluble drugs has been suggested to involve solubilization of drug in bile salt and phospholipid mixed micelles (Porter et al., 2008). If these micellar solutions significantly increase the solubility of a poorly water-soluble drug, then the release of bile salt/phospholipid at the time of dosing could improve bioavailability substantially. Table 3 summarized the solubility of TAL in bile salt/ phospholipid solutions (FaSSIF and FeSSIF). The solubilization of TAL in water, FaSSIF and FeSSIF solution was investigated. It can be seen from the result that the presence of bile salt/phospholipid increased the solubility of TAL from 0.23 (solubility in water) 2.16 mg/ml (solubility in FaSSIF) and further increased to 4.53 mg/mL due to higher concentrations of bile salt/phospholipid in FeSSIF. The solubility increased in presence of higher bile salt/phospholipid concentration indicates that TAL would be benefited during digestion in the gut under fed/fasted conditions.

The combination of Imwitor 308 as oily phase with Transcutol (TC) (1:1) and HCO-30 as surfactant with an equal ratio (F6) showed the higher solubility in both anhydrous and 99% diluted formulations and yielded a transparent clear appearance. On the other hand, Capmul MCM (CMCM) and TC with HCO-30 as surfactant (AF6) have high equilibrium solubility in both anhydrous and 99% diluted formulation and also produced a transparent clear appearance. TC, which is a solubilizer and absorption enhancer, was found to be a very efficient solubilizer of TAL (AF7), and was chosen for dissolution studies.

The data from the 99% diluted formulations in Table 3 can be used to estimate whether or not the solubility of TAL would be exceeded or supersaturated on dilution of each formulation with water. Using these data, the drug can be dissolved at a lower saturation level to avoid any precipitation upon aqueous dilution. For example, if TAL was dissolved at 80% solubility in the anhydrous formulations, its solubility limit will not be exceeded for a sufficient period of time as dilution proceed and the drug may remain in solution. From the overall results, the formulations F2, F6, AF6, and AF7 with higher drug solubility and better aqueous dispersibility (Bluish/Transparent appearance) were chosen for the dissolution studies.

### *In vitro* Dissolution Studies

**FIGURE 5:**
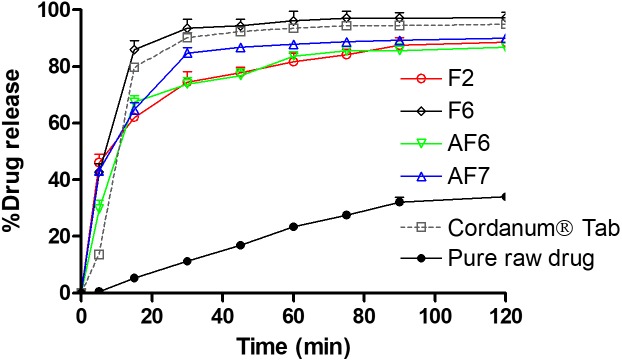
*In vitro* release of TAL from the pure raw TAL powder, marketed product (Cordanum^®^) and the representative self-nanoemulsifying formulations in acidic media (pH 1.2) at 37°C. The formulations represent (F2) CoFA:I988(7:3)/TO106V(1/1), (F6) I308:TC(1:1)/HCO30(1/1), (AF6) CMCM:TC(1:1)/HCO30(1/1) and (AF7) TC. Data are represented as mean ± SD, *n* = 3.

**FIGURE 6:**
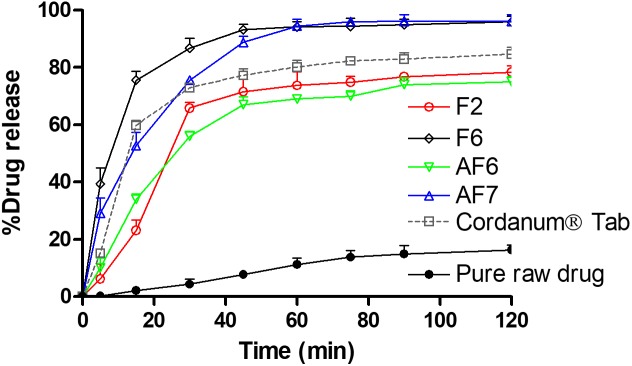
*In vitro* release of TAL from the pure raw TAL powder, marketed product (Cordanum^®^) and the representative self-emulsifying formulations in (pH 6.8) at 37°C. The formulations represent (F2) CoFA:I988(7:3)/TO106V(1/1), (F6) I308:TC(1:1)/HCO30(1/1), (AF6) CMCM:TC(1:1)/HCO30(1/1) and (AF7) TC. Data are represented as mean ± SD, *n* = 3.

The dissolution studies were performed for various SNEDDS (i.e., F2, F6, AF6, AF7) and the commercial tablet Cordanum^®^. The release of TAL from the F2, F6, and AF7 reached 45% within 5 min versus 13 and 0.5% release from the commercial Cordanum^®^ tablet and pure raw powder in the acidic medium (*p* < 0.001). Among all the formulations, F6 showed a significant release of TAL (approximately 98%, *p* < 0.001) after 120 min (Figure 5). In the higher pH medium F2 and AF7 released approximately 80 and 96% of TAL, respectively, and F2 released 83% within 75 min. The commercial tab (Cordanum^®^) and pure raw powder released 81 and 14% of TAL in 75 min respectively (*p* < 0.001) (Figure 6). The overall dissolution studies showed that F6 formulation has a comparably higher TAL release compared to other representative formulations, Cordanum^®^ tablet and pure raw drug powder. Formulation F6 was chosen for further pharmacokinetic studies.

It is common that weakly base compounds (pKa > 8) have high solubility in acidic conditions (stomach media) and low in neutral and or basic conditions (intestinal media), as they can be converted into uncharged molecule in neutral and or basic conditions (Shahba et al., 2016). Therefore, basic drugs might dissolve completely in the stomach, maintain drug in solubilized form and later precipitate in the intestine due to sudden changes in pH (pH shift to higher) and/or extensive dilution of excipients. However, the purpose should be to avoid precipitation and maintain the drug in dissolved state in both acidic and neutral/basic media, which will ensure the complete oral absorption of the poorly soluble basic drug compounds. In the present study the developed SNEDDS were able to improve the solubility and dissolution of TAL at pH 1.2 and 6.8, thus more likely to enhance its absorption in gastrointestinal tract (GIT).

### Analysis of *in vitro* Post-digested Products

**FIGURE 7:**
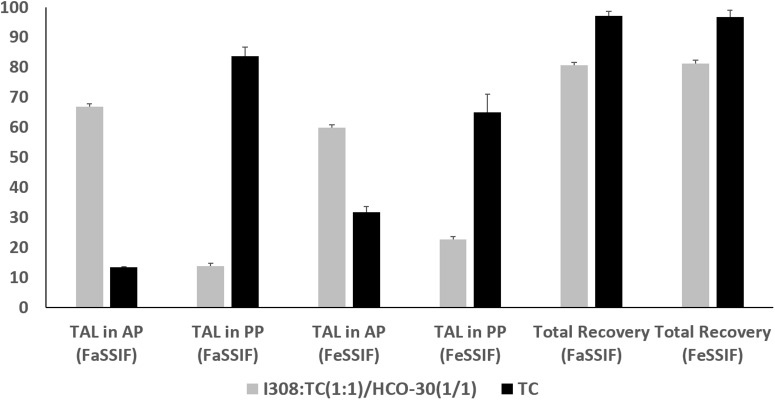
The % TAL solubilized in aqueous phase (AP), pellet phase (PP) and total recovery after lipolysis using fed (FeSSIF) and fasted (FaSSIF) media. The formulations represent (F6) I308:TC (1:1)/HCO30 (1/1) and (AF7) TC. Data are represented as mean ± SD, *n* = 3.

The *in vitro* lipolysis tests in a small scale was performed in the current study to understand the *in vivo* behavior of the representative SNEDDS. TAL distribution across the digested phases of the representative lipid formulations under fed and fasted conditions are depicted in Figure 7. The data showed that Type IIIB formulations [I308:TC (1:1)/HCO-30(1/1)] presented comparably higher amount of drug in aqueous phase (AP)both under fed and fasted (59.81 and 66.83%) conditions, although around 80% of the total drug was recovered after 30 min digestion. In contrast, Type IV formulation (extremely hydrophilic, TC) showed only 31.77 and 13.41% drug in AP under fed and fasted conditions after 30 min digestion period from the 97% of total drug recovered (Figure 7).

The overall results suggested that the inclusion of monoglycerides (I308) and surfactant (HCO-30) in the formulation produced fine emulsion on dilution with simulated BS/PL solution and increased the rate of digestion. Therefore, the digestion of this formulation, I308:TC (1:1)/HCO-30(1/1) results high amount of drug in AP comparably with Type IV (lipid free) formulation of TC. Type IV formulation systems have high TAL solubility which may digest well but retain majority of the drug in the pellet phase after digestion as precipitates.

The biorelevant *in vitro* lipolysis model (high physiological relevance) is the key to generate high quality human reference data from the investigation of drug release and absorption of lipid formulation from the GI lumen. During *in vitro* lipolysis studies, the fate of the drug can be determined (whether it is solubilized or precipitated) from the products after completion of the reaction of lipolysis.

### *Ex vivo* Intestinal Permeation

**FIGURE 8:**
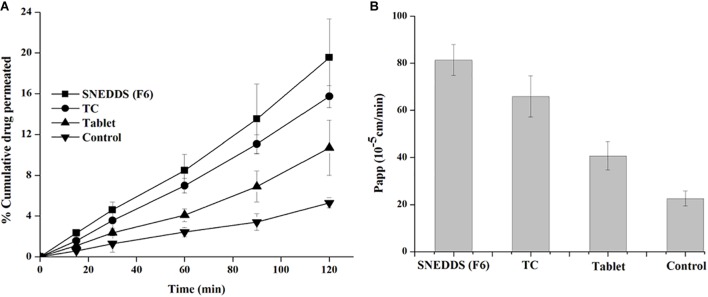
Apparent permeability of TAL (**A**: % cumulative drug concentration permeated and **B**: apparent permeability of coefficient) from the SNEDDS (F6), TC (AF7), pure TAL powder (control) and Cordanum^®^ suspensions (marketed tablet) using non-everted rat intestinal sac model.

The non-everted rat intestinal sac is significantly conducted to confirm the results obtained from the *in vitro* release studies and predict *in vivo* absorption of TAL. This experiment provides the benefit of reduction of effort and experimental costs compared with *in vivo* animal studies (Zhang et al., 2014). The non-everted sac method was used to evaluate *ex vivo* intestinal permeation of the selected SNEDDS compared to the pure TAL (control) and marketed tablets. Figure 8A displays the cumulative permeated concentration of TAL over a period of 2 h. The increased permeated TAL from the SNEDDS (F6) was higher than that permeated from TC (AF7), control and tablet suspensions. Moreover, *ex vivo* permeation data were in agreement with the results of the *in vitro* release studies. The small intestine permeation of TAL-loaded SNEDDS established up to 20% (F6) in 2 h, whereas exhibited a maximum of 16, 11, and 5% from TC, tablet suspension and control, respectively (Figure 8A).

A TAL-loaded SNEDDS produced a Papp up to 81.3 × 10^-5^ cm/sec for SNEDDS (F6) at 2 h, whereas TAL exhibited a maximum of 65.8 × 10^-5^, 40.7 × 10^-5^, and 5.9 × 10^-5^ cm/s from TC formulation, tablet and powder suspension (control), respectively (Figure 8B). It was observed that a SNEDDS composed of I308:TC/CO30(1/1) in the ratio of 1:1, respectively, showed a significant increase in drug rat gut diffusion. There are different mechanisms could explain the increase intestinal absorption of TAL from SNEDDS formulation. The high permeation might be attributed to the rapid dissolution of TAL in the intestinal sac and a subsequent rapid diffusion. It is supposed that the higher drug absorption is due to the resultant nano-emulsion droplet size from SNEDDS in the intestinal region, which could enhance absorption of TAL (Zhang et al., 2014). Thus, nanoemulsion provides a larger surface area available for permeation of the drug through the intestinal membrane. Moreover, the high drug solubility and ability of self-emulsification of the formulation F6 may have contributed to the increased intestinal absorption of TAL. The bio-enhancing activities of the ingredients used in SNEDDS (F6), which contains I308 as surfactant that improve the permeability by disturbing the lipids of the cell membrane. A similar correlation was observed with *in vitro* drug release studies (Figure 5, 6).

### *In vitro* Hemolysis Test

**FIGURE 9:**
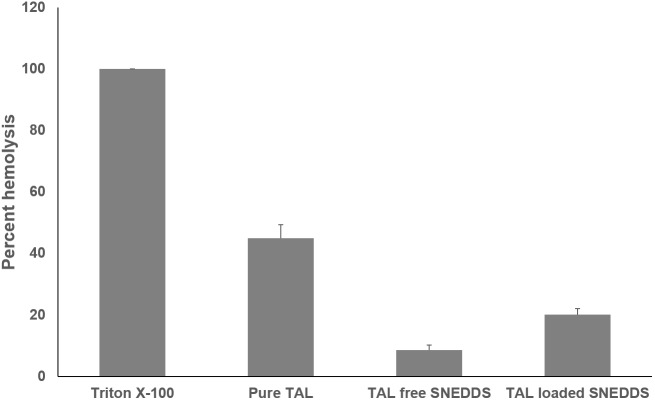
*In vitro* toxicity test of pure TAL, Blank SNEDDS (drug free), and TAL loaded SNEDDS on blood erythrocytes. The extent of damage caused to blood erthyrocytes by TAL was measured as percentage lysis of total (RBC) red blood cells added. The data is a mean ± SD of three set of different experiments. Formulation represents F6 SNEDDS [I308:TC(1:1)/HCO30(1/1)].

The effect of F6 SNEDDS formulation to cause RBC toxicity was studied by *in vitro* RBC lysis (hemolysis) test. 100% lysis of RBC caused by Triton X-100 was used as positive control. Figure 9 showed the percentage RBC lysis induced by the representative SNEDDS formulation. In comparison with pure TAL powder and blank F6 (drug free) SNEDDS, insignificant RBC lysis was caused by TAL loaded F6 SNEDDS.

### *In vivo* Bioavailability Studies

**FIGURE 10:**
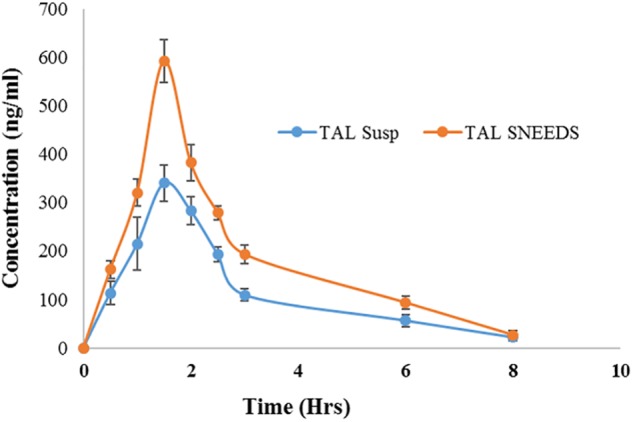
Plasma concentration– time profiles of TAL after a single oral administration of SNEDDS formulation [I308: TC (1:1)/HCO-30 (1/1)] and TAL powder to rats at a dose equivalent to 10 mg/kg TAL (mean ± SEM, *n* = 6).

The pharmacokinetics of SNEDDS [F6, I308: TC (1:1)/HCO-30 (1/1)] containing TAL confirmed the enhancement of bioavailability of the drug (Figure 10 and Table 4). The *C*_max_ of TAL raw powder after oral administration was 340.75 ± 7.80 ng/mL and the T_max_ was 1.5 h, while in the oral administration of representative SNEDDS formulation of TAL, the C _max_ and T _max_ were 593.05 ± 6.04 ng/mL and 1.5 h, respectively. The *C*_max_ value of TAL from the SNEDDS formulation was significantly increased (74.04%, *p* < 0.0001). The AUC_0-_*t* of TAL was also significantly increased in the SNEDDS-treated group as compared to the only TAL powder-treated group (58.33%, *p* < 0.0001), from 927.35 ± 10.02 to 1468.23 ± 18.52 ng h/mL, respectively. The increase in relative bioavailability was found to be 158.33%. The calculated oral clearance was significantly declined (95.95%, *p* < 0.05) from 0.15 ± 0.34 to 0.01 ± 0.0001 mL/kg, while the estimated oral volume of distribution at steady state was significantly decreased (95.70%, *p* < 0.001) from 0.37 ± 0.84 to 0.02 ± 0.001 mL/kg.

**Table 4:** Comparison of pharmacokinetic parameters of TAL after oral administration of TAL powder and SNEDDS formulation [F6, I308: TC (1:1)/HCO-30 (1/1)] containing TAL.

Parameter	Unit	TAL Powder	± SEM	TAL SNEDDS	± SEM	% Control
Lambda_z	1/h	0.39	0.03	0.41	0.03	6.11
t1/2	h	1.81	0.17	1.70	0.18	6.00
Tmax	h	1.50	0.00	1.50	0.00	0.00
Cmax	ng/ml	340.75***	27.80	593.05***	36.99	74.04
AUC 0-t	ng/ml^∗^h	927.35***	56.02	1468.23***	78.29	58.33
AUC 0-t/0-inf_obs		0.94	0.01	0.96	0.01	1.55
MRT 0-inf_obs	H	3.16	0.34	3.06	0.67	3.10
Vz/F_obs	(mg)/(ng/ml)	0.37**	0.84	0.02**	0.001	95.70
Cl/F_obs	(mg)/(ng/ml)/h	0.15*	0.39	0.01*	0.0001	95.594


*^∗^Data are expressed as mean ± SEM (*n* = 6), ^∗^*p* < 0.05, ^∗∗^*p* < 0.001, and ^∗∗∗^*p* < 0.0001. T_*max*_, time of peak concentration; C_*max*_, peak of maximum concentration; AUC_*0*→*t*_, area under the concentration time profile curve up to time 24 h; AUC_*0*→∞_, area under the concentration time profile curve extrapolated to infinity; K_*el*_, elimination rate constant; V_*ss*_, volume of distribution at steady state; T_*1*/*2*_, half-life; MRT, mean residence time; CL/F, oral clearance.*

The improvement in bioavailability of TAL from SNEDDS formulation may be due to improved solubility and dissolution profiles of TAL. The enhanced oral bioavailability of TAL may also be due to the faster uptake of the nanoemulsion resulting from SNEDDS formulation by enterocytes at the absorption site (Elgart et al., 2013). There are several reports which confirm this view (Zhang et al., 2014, 2015; Mohsin et al., 2016).

## Conclusion

The developed SNEDDS formulations showed a high solubility and dissolution profile of TAL in the anhydrous, 99% diluted formulations and maintained solubilized TAL mostly in aqueous phase of the digested samples *in vitro.* The gut permeability and oral bioavailability of the representative SNEDDS formulation was found to increase by 4- and 1.58-fold when compared to pure raw drug, respectively. The overall results conclude that LFCS Type IIIB lipid-based SNEDDS can be explored as a potential vehicle to obtain higher drug loading, improved dissolution, digestion, permeation and enhanced bioavailability of the poorly soluble drug, TAL with no toxic effects.

## Ethics Statement

All experimental procedures of the animal studies were conducted in accordance with the National Institutes of Health Guide for the Care and Use of Laboratory Animals (NIH Publications No. 80–23; 1996) as well as the animal facilities guidelines approved from the Ethical committee of Experimental Animal Care Center, College of Pharmacy, King Saud University (Clearance No. 5698).

## Author Contributions

MK performed formal experiments in the study, obtained funding and prepared the original draft of the manuscript. MA-S, AA, MR, and MH provided technical support and analyzed the *in vivo* data. MB conducted the intestinal permeability experiments and revised the entire manuscript. AK and AMA investigated RBC toxicity and revised the manuscript. FA and MH contributed to editing and critically revising the manuscript for important intellectual content. All co-authors approved the final version of the manuscript for submission.

## Conflict of Interest Statement

The authors declare that the research was conducted in the absence of any commercial or financial relationships that could be construed as a potential conflict of interest.

## References

[B1] Awadallah B. Schmidt P. C. Holzgrabe U. Wahl M. A. (2003). Quantitation of talinolol and other beta-blockers by capillary electrophoresis for in vitro drug absorption studies. *Electrophoresis* 24 2627–2632. 10.1002/elps.200305443 12900875

[B2] Chiou W. L. Ma C. Chung S. M. Wu T. C. (2001). An alternative hypothesis to involvement of intestinal P-glycoprotein as the cause for digoxin oral bioavailability enhancement by talinolol. *Clin. Pharmacol. Ther.* 69 79–81. 11180042

[B3] Craig D. Q. M. Barker S. A. Banning D. Booth S. W. (1995). An investigation into the mechanisms of self-emulsification using particle size analysis and low frequency dielectric spectroscopy. *Int. J. Pharm.* 114 103–110. 10.1016/0378-5173(94)00222-q

[B4] Devraj R. Williams H. D. Warren D. B. Mohsin K. Porter C. J. Pouton C. W. (2013). In vitro assessment of drug-free and fenofibrate-containing lipid formulations using dispersion and digestion testing gives detailed insights into the likely fate of formulations in the intestine. *Eur. J. Pharm. Sci.* 49 748–760. 10.1016/j.ejps.2013.04.036 23684915

[B5] Elgart A. Cherniakov I. Aldouby Y. Domb A. J. Hoffman A. (2013). Improved oral bioavailability of BCS class 2 compounds by self nano-emulsifying drug delivery systems. (SNEDDS): the underlying mechanisms for amiodarone and talinolol. *Pharm. Res.* 30 3029–3044. 10.1007/s11095-013-1063-y 23686373

[B6] Fatouros D. G. Karpf D. M. Nielsen F. S. Mullertz A. (2007). Clinical studies with oral lipid based formulations of poorly soluble compounds. *Ther. Clin. Risk Manag.* 3 591–604. 18472981 PMC2374933

[B7] Galia E. Nicolaides E. Horter D. Lobenberg R. Reppas C. Dressman J. B. (1998). Evaluation of various dissolution media for predicting in vivo performance of class I and II drugs. *Pharm. Res.* 15 698–705. 9619777 10.1023/a:1011910801212

[B8] Gershanik T. Benita S. (2000). Self-dispersing lipid formulations for improving oral absorption of lipophilic drugs. *Eur. J. Pharm. Biopharm.* 50 179–188. 10.1016/s0939-6411(00)00089-8 10840200

[B9] Ghai D. Sinha V. R. (2012). Nanoemulsions as self-emulsified drug delivery carriers for enhanced permeability of the poorly water-soluble selective beta(1)-adrenoreceptor blocker Talinolol. *Nanomedicine* 8 618–626. 10.1016/j.nano.2011.08.015 21924224

[B10] Gramatte T. Oertel R. Terhaag B. Kirch W. (1996). Direct demonstration of small intestinal secretion and site-dependent absorption of the beta-blocker talinolol in humans. *Clin. Pharmacol. Ther* 59 541–549. 10.1016/s0009-9236(96)90182-4 8646825

[B11] Iglicar P. Legen I. Vilfan G. Selic L. Prezelj A. (2009). Permeability of a novel beta-lactamase inhibitor LK-157 and its ester prodrugs across rat jejunum in vitro. *J. Pharm. Pharmacol.* 61 1211–1218. 10.1211/jpp/61.09.0011 19703371

[B12] Kale A. A. Patravale V. B. (2008). Design and evaluation of self-emulsifying drug delivery systems. (SEDDS) of nimodipine. *AAPS PharmSciTech* 9 191–196. 10.1208/s12249-008-9037-9 18446481 PMC2976914

[B13] Kang B. K. Lee J. S. Chon S. K. Jeong S. Y. Yuk S. H. Khang G. (2004). Development of self-microemulsifying drug delivery systems. (SMEDDS) for oral bioavailability enhancement of simvastatin in beagle dogs. *Int. J. Pharm.* 274 65–73. 10.1016/j.ijpharm.2003.12.028 15072783

[B14] Kazi M. Al-Qarni H. Alanazi F. K. (2017). Development of oral solid self-emulsifying lipid formulations of risperidone with improved in vitro dissolution and digestion. *Eur. J. Pharm. Biopharm.* 114 239–249. 10.1016/j.ejpb.2017.01.015 28159721

[B15] Khan A. A. Husain A. Jabeen M. Mustafa J. Owais M. (2012). Synthesis and characterization of novel n-9 fatty acid conjugates possessing antineoplastic properties. *Lipids* 47 973–986. 10.1007/s11745-012-3707-9 22923370

[B16] Kommuru T. R. Gurley B. Khan M. A. Reddy I. K. (2001). Self-emulsifying drug delivery systems. (SEDDS) of coenzyme Q10: formulation development and bioavailability assessment. *Int. J. Pharm.* 212 233–246. 10.1016/s0378-5173(00)00614-1 11165081

[B17] Mohsin K. Alamri R. Ahmad A. Raish M. Alanazi F. K. Hussain M. D. (2016). Development of self-nanoemulsifying drug delivery systems for the enhancement of solubility and oral bioavailability of fenofibrate, a poorly water-soluble drug. *Int. J. Nanomed.* 11 2829–2838. 10.2147/IJN.S104187 27366063 PMC4914069

[B18] Mohsin K. Alanazi F. (2012). The fate of paclitaxel during in vitro dispersion testing of different lipid-based formulations. *J. Drug Deliv. Sci. Technol.* 22 197–204. 10.1016/s1773-2247(12)50026-2

[B19] Mohsin K. Long M. A. Pouton C. W. (2009). Design of lipid-based formulations for oral administration of poorly water-soluble drugs: precipitation of drug after dispersion of formulations in aqueous solution. *J. Pharm. Sci.* 98 3582–3595. 10.1002/jps.21659 19130605

[B20] Pathak S. M. Musmade P. B. Bhat K. M. Udupa N. (2010). Validated HPLC method for quantitative determination of talinolol in rat plasma and application to a preclinical pharmacokinetic study. *Bioanalysis* 2 95–104. 10.4155/bio.09.162 21083123

[B21] Porter C. Pouton C. Cuine J. Charman W. (2008). Enhancing intestinal drug solubilisation using lipid-based delivery systemsâ˜†. *Adv. Drug Deliv. Rev.* 60 673–691. 10.1016/j.addr.2007.10.014 18155801

[B22] Pouton C. Porter C. (2008). Formulation of lipid-based delivery systems for oral administration: materials, methods and strategiesâ˜†. *Adv. Drug Deliv. Rev.* 60 625–637. 10.1016/j.addr.2007.10.010 18068260

[B23] Pouton C. W. (1985). Self-emulsifying drug delivery systems: assessment of the efficiency of emulsification. *Int. J. Pharm.* 27 335–348. 10.1016/0378-5173(85)90081-x

[B24] Pouton C. W. (2000). Lipid formulations for oral administration of drugs: non-emulsifying, self-emulsifying and ‘self-microemulsifying’ drug delivery systems. *Eur. J. Pharm. Sci.* 11(Suppl. 2), S93–S98. 11033431 10.1016/s0928-0987(00)00167-6

[B25] Raish M. Ahmad A. Ansari M. A. Alkharfy K. M. Ahad A. Khan A. (2019). Effect of sinapic acid on aripiprazole pharmacokinetics in rats: possible food drug interaction. *J. Food Drug Anal.* 27 332–338. 10.1016/j.jfda.2018.06.002 30648588 PMC9298613

[B26] Schwarz U. I. Gramatte T. Krappweis J. Oertel R. Kirch W. (2000). P-glycoprotein inhibitor erythromycin increases oral bioavailability of talinolol in humans. *Int. J. Clin. Pharmacol. Ther.* 38 161–167. 10.5414/cpp38161 10783825

[B27] Shahba A. A. Mohsin K. Abdel-Rahman S. I. Alanazi F. K. (2017). Solidification of cinnarizine self-nanoemulsifying drug delivery systems by fluid bed coating: optimization of the process and formulation variables. *Pharmazie* 72 143–151. 10.1691/ph.2017.6089 29442049

[B28] Shahba A. A. Mohsin K. Alanazi F. K. (2012). Novel self-nanoemulsifying drug delivery systems. (SNEDDS) for oral delivery of cinnarizine: design, optimization, and in-vitro assessment. *AAPS PharmSciTech* 13 967–977. 10.1208/s12249-012-9821-4 22760454 PMC3429665

[B29] Shahba A. A.-W. Mohsin K. Alanazi F. K. Abdel-Rahman S. I. (2016). Optimization of self-nanoemulsifying formulations for weakly basic lipophilic drugs: role of acidification and experimental design. *Braz. J. Pharm. Sci.* 52 653–667. 10.1590/s1984-82502016000400009

[B30] Shakeel F. Haq N. Alanazi F. K. Alsarra I. A. (2016). Surface-adsorbed reverse micelle-loaded solid self-nanoemulsifying drug delivery system of talinolol. *Pharm. Dev. Technol.* 21 131–139. 10.3109/10837450.2014.971379 25318634

[B31] Siegmund W. Ludwig K. Engel G. Zschiesche M. Franke G. Hoffmann A. (2003). Variability of intestinal expression of P-glycoprotein in healthy volunteers as described by absorption of talinolol from four bioequivalent tablets. *J. Pharm. Sci.* 92 604–610. 10.1002/jps.10327 12587122

[B32] Strickley R. G. (2004). Solubilizing excipients in oral and injectable formulations. *Pharm. Res.* 21 201–230. 10.1023/b:pham.0000016235.32639.23 15032302

[B33] Terhaag B. Palm U. Sahre H. Richter K. Oertel R. (1992). Interaction of talinolol and sulfasalazine in the human gastrointestinal tract. *Eur. J. Clin. Pharmacol.* 42 461–462. 1355428 10.1007/BF00280137

[B34] Trausch B. Oertel R. Richter K. Gramatte T. (1995a). Disposition and bioavailability of the beta 1-adrenoceptor antagonist talinolol in man. *Biopharm. Drug Dispos.* 16 403–414. 10.1002/bdd.2510160505 8527689

[B35] Trausch B. Oertel R. Richter K. Gramatte T. (1995b). The protein binding of talinolol. *Pharmazie* 50:72. 7886130

[B36] Weng T. Qi J. Lu Y. Wang K. Tian Z. Hu K. (2014). The role of lipid-based nano delivery systems on oral bioavailability enhancement of fenofibrate, a BCS II drug: comparison with fast-release formulations. *J. Nanobiotechnol.* 12:39. 10.1186/s12951-014-0039-3 25248304 PMC4180958

[B37] Wetterich U. Spahn-Langguth H. Mutschler E. Terhaag B. Rosch W. Langguth P. (1996). Evidence for intestinal secretion as an additional clearance pathway of talinolol enantiomers: concentration- and dose-dependent absorption in vitro and in vivo. *Pharm. Res.* 13 514–522. 8710739 10.1023/a:1016029601311

[B38] Zhang J. Li J. Ju Y. Fu Y. Gong T. Zhang Z. (2015). Mechanism of enhanced oral absorption of morin by phospholipid complex based self-nanoemulsifying drug delivery system. *Mol. Pharm.* 12 504–513. 10.1021/mp5005806 25536306

[B39] Zhang X. Chen G. Zhang T. Ma Z. Wu B. (2014). Effects of PEGylated lipid nanoparticles on the oral absorption of one BCS II drug: a mechanistic investigation. *Int. J. Nanomed.* 9 5503–5514. 10.2147/IJN.S73340 25473287 PMC4251747

